# A language to analyze, describe, and explore collections of visual art

**DOI:** 10.1186/s42492-021-00071-3

**Published:** 2021-03-01

**Authors:** Hermann Pflüger

**Affiliations:** Institute for Visualization and Interactive Systems, University of Stuttgart, 70569 Stuttgart, Germany

**Keywords:** Collections of visual art, Visual computing, Personalized digital libraries, Digital humanities, Semantics

## Abstract

A vast quantity of art in existence today is inaccessible to individuals. If people want to know the different types of art that exist, how individual works are connected, and how works of art are interpreted and discussed in the context of other works, they must utilize means other than simply viewing the art. Therefore, this paper proposes a language to analyze, describe, and explore collections of visual art (LadeCA). LadeCA combines human interpretation and automatic analyses of images, allowing users to assess collections of visual art without viewing every image in them. This paper focuses on the lexical base of LadeCA. It also outlines how collections of visual art can be analyzed, described, and explored using a LadeCA vocabulary. Additionally, the relationship between LadeCA and indexing systems, such as ICONCLASS or AAT, is demonstrated, and ways in which LadeCA and indexing systems can complement each other are highlighted.

**Video abstract.**

**Supplementary Information:**

The online version contains supplementary material available at 10.1186/s42492-021-00071-3.

## Introduction

The number of digital images is constantly growing, and there are currently billions of images available. Flickr (www.flickr.com) alone hosts several billion images. Even when restricted to well-structured and documented collections of digital images with science-based metadata and annotations, there are still millions of images available. For example, Getty Images (www.gettyimages.com) hosts 80 million images, akg-images (www.akg-images.de) hosts more than 10 million images in the genres of art, culture, history, politics, sciences, and media, and the New York Public Library’s website (www.nypl.org) hosts one million images. In terms of only art, there are collections containing several hundred thousand images that are available; for instance, the Albertina Wien (www.albertina.at) has almost 900,000 sheets of prints pasted into historical volumes known as “Klebealben”, which are currently being digitalized in high resolution. However, it is not just the vast volume of images that makes it impossible for an individual researcher to gain an overview of the entire art world; the different relationships among the art works present a further complexity. For example, art experts W. Grasskamp, C. Lange, and J. Voss claimed in a 2015 radio interview [1, 2] that the increasing quantity of art works and growing complexity of the relations among art works has led to a situation where the original tasks of museums (i.e., preserving, collecting, researching, and teaching [3]) can no longer be completed in conventional ways. Moreover, Walter Grasskamp stated in an interview that an art expert would have to visit nine exhibitions a day in order to cover all the exhibitions that are held in Germany alone. As this is not practically feasible, when assessing art, art experts have not actually seen most existing art; it is, so to speak, “invisible” to them.

There are personalized digital archives that go beyond the primary purpose of making individual or groups of images from the field of visual art available [4]. Recent browser developments have aimed to help people find images in large collections or to examine and compare images and image groups [5–7]. In contrast, the proposed LadeCA language, which allows users to analyze, describe, and explore collections of visual art, focuses less on individual images and instead focuses on image sets and their properties to make large image sets accessible as a whole.

**Fig. 1:**
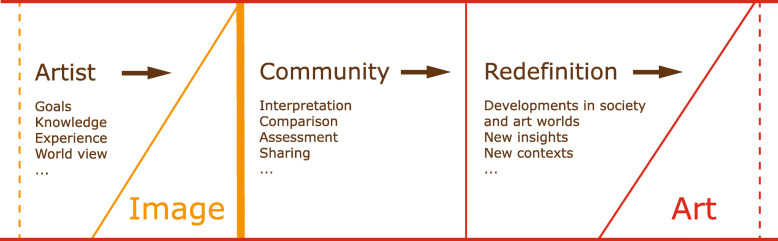
Definition of art

A language about art collections must be based on a definition of art that determines the activities people must perform with art collections. Guided by definitions in works such as refs. [8–13], in this study, art was defined as a process in which an artist and a community collaborate, as illustrated graphically in Fig. 1. In the first stage, an artist creates, say, a drawing or painting guided by their goals, experience, knowledge, and world view but without relying solely on known techniques or given contents. In the second stage, the result must be accepted as art by a community that appreciates the work as art and discusses it in an artistic context. In the final stage, the work must be embedded in the historical process of art. As the above definition suggests, art is a dynamic process in which the art object itself forms the initial step. The decisive elements in this process are analyses, structuring, and interpretations of the object. The decision of whether an image is a work of art or not is determined by this process.

By its inherent nature, visual art is aimed directly at human visual perception, and a language determined by art must be able to express the way in which art affects people and what associations different works of art have with a given piece of art. The context in which art is viewed and analyzed is also crucial to the concept of art. LadeCA uses algorithms that can automatically determine visual relationships similarly to human perception. Nonvisual relationships are considered via the interaction between users and algorithms and by using metadata. Thus, as described in more detail later in this paper, LadeCA can formulate statements about sets of images (without any restriction on the type of artwork) on a semantic level and support the underlying process of art outlined in Fig. 1. Above all, LadeCA can handle the vast and varied distribution of digital images and the complex relationships that arise from the manifold compilations of images in collections and exhibitions.

**Fig. 2:**
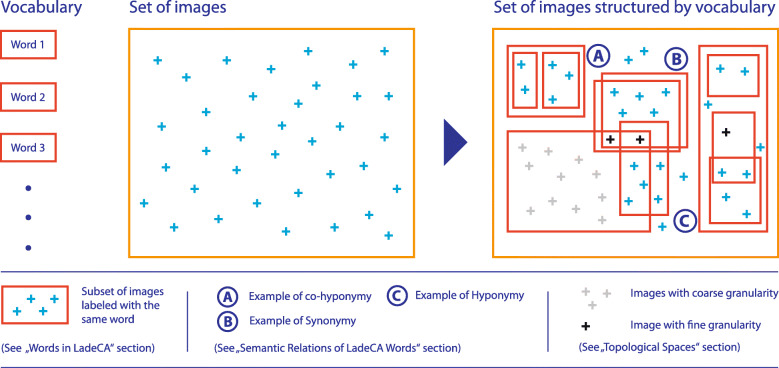
Schematic representation of how a vocabulary structures a set of images

The basic elements of LadeCA are related images that each represents a unit of meaning. These elements are the words of LadeCA, which are defined by users providing typical images for those words; these images are called the prototypes. With the prototypes of a particular word, LadeCA automatically creates a special classifier for that word. The classifier can then automatically determine whether a given image can be labeled with the corresponding word. Several words result in a vocabulary, and LadeCA can automatically structure any set of images. These structures can be interpreted automatically or by the user, which leads to statements about a given set of images. Figure 2 illustrates how a LadeCA vocabulary automatically structures sets of images.

This paper focuses on the lexical base of LadeCA. A lexical base represents how words are created and includes their properties and how they are semantically related. This paper also outlines how sets of images can be analyzed, described, and explored using a LadeCA vocabulary. Additionally, the relationship between LadeCA and indexing systems, such as ICONCLASS [14] and AAT [15], is demonstrated, and ways in which LadeCA and indexing systems can complement each other are highlighted. Furthermore, it is shown that all the methods and algorithms required to use LadeCA are available and suitable for interactive use, even if they are applied to large collections, even those containing one million images.

## Related work

In this section, current available means for working with digital libraries and collections of visual art are presented. The major advancements under discussion and new methods that are currently being developed in this field are discussed. In view of current possibilities and subjects of discussion, about the novelties of LadeCA are summarized to highlight its contribution to research on digital libraries and collections of visual art. LadeCA’s main contributions are twofold: its methods of handling collections of art and its new technical methods, which enable its practical realization.

Many museums nowadays offer access to their images via digital databases, including the British Museum (www.britishmuseum.org), Rijksmuseum (www.rijksmuseum.nl), and the Metropolitan Museum of Art (www.metmuseum.org). Other institutions of various kinds also offer their digital image databases of visual art, which are sometimes considerably larger than those of actual museums, for research purposes, such as Getty Images (www.gettyimages.de), akg-images (www.akg-images.de), and prometheus (www.prometheus-bildarchiv.de). In all of these image databases, a user can search for images with the aid of metadata (e.g., an artist, theme, or object type). In the abovementioned museums, many of the images are open access and can be downloaded in high resolution. Additional images that are related to a particular image, i.e., images with the same metadata, are also often provided. In this manner, a user can obtain information about individual images and collate small samples; this is the current situation for publicly accessible image databases. This type of image search has the decisive disadvantage that only images that are somehow known to the researcher can be found, i.e., some associated metadata must already be known to the researcher. It should be specifically emphasized here that one cannot determine and present properties of large sets of images as a whole with this research approach.

Meanwhile, there have been approaches that use search strategies based on the actions of individual users or groups in order to generalize their search queries and structure databases in a suitable manner (e.g., see refs. [16–18]). However, structuring based on user actions can also only be based on some (implicitly) existing knowledge from one of the users, thereby increasing in particular (incorrect) assessments, which in turn leads to so-called filter bubbles.

Indexing systems such as ICONCLASS [14] and AAT [15] are systems of knowledge organization for capturing and indexing image content. Indexed databases can be searched for specific images in the same manner as metadata searching described above; however, a search via an indexing system, in particular, a semantic-level search, offers much finer search criteria. Indexing systems usually have a hierarchical structure, and relationships between the individual classes are often formulated by experts (e.g., AAT). Indexed databases can therefore be structured automatically, i.e., relationships between individual images can be determined automatically, as can clusters of (partially) synonymous images (“word fields”), and structures can be determined based on hyponymy (see “Semantic relations of LadeCA words” section for definition). This results in semantic networks (without filter bubbles), which allows a user to describe, evaluate, and present the structure of image sets [17, 19–22]. This in turn enables software developers to create interfaces that allow users to recognize the content and properties of image databases and thus provide them with a more powerful framework for image research than is offered by conventional databases. The difficulty in creating and using indexing systems is that it must be done manually and with expert knowledge (Indexing systems section). As a result, indexing systems are a powerful means for image research, but they are also very complex to create and therefore become static and inflexible. The latter properties are in contrast to the flexible and constantly changing way in which images are interpreted; this roughly corresponds to the problem of the interpretation of natural language [23–25] and, more generally, of the use of symbols in interaction among humans [9, 10].

In some respects, the Mnemosyne Atlas projected by Aby Warburg (1866–1929) [26] suggests a solution to the problem described above. Aby Warburg formed units of meaning, i.e., words, by grouping images and arranged these words spatially such that the proximity of the words in space reflects the relationship between the words. This leads to an atlas of related words, which roughly corresponds to a semantic network. However, Warburg considered the words, defined by groups of images, and their spatial arrangement as flexible. Given the means available at the time, Wartburg could of course not implement his concept in a practical manner. This brings us to this research because, in some way, LadeCA can be seen as an extension and realization of the Mnemosyne Atlas with visual computing and flexible semantic networks.

To consider LadeCA as a modern implementation of the Mnemosyne Atlas, the first step is to create image groups as words. This process can be supported by searching for images related to a given image using suitable interfaces. In this manner, related images can be found effectively and reliably from a basis set. Bell et al. [27] presented a procedure, which is mainly based on automatic object recognition in images; however, for interactive use alongside large number of images, the computation times of the typical object recognition methods used in that work were too long. Pflüger et al. [28] presented a method for searching related images in large sets of images. The computing time of this method is sufficiently fast for the given application, but the method is relatively experimental and does not consider information given by metadata/annotations.

The main weakness of the Mnemosyne Atlas concept is that a word is defined exclusively by all images that belong to that word. As a result, a word is determined not only by suitable sample images but by all images belonging to the word, and it is only defined for a given basis of images. Words given in this manner largely elude algorithmic analysis of relationships between words for analyzing image sets and creating semantic networks.

Therefore, the main tasks in developing LadeCA in the spirit of the Mnemosyne Atlas were to develop suitable algorithms for group formation when creating words and, in particular, to enable the creation of classifiers as intentional definitions of words that allow algorithmic analysis (Semiotic triangle section). The challenge in developing appropriate algorithms was that they should all be used within the users’ interactive word formation processes, so they had to be real-time capable.

The bases for the required algorithms are comparison functions that can be used to estimate the degree of relationship between images; these are often seen as similarity measures. The development of comparison functions is a well-researched area. However, there is no universally applicable function, as there are infinite types of image relationships (visual similarity, similarity of objects, style, time of creation, artists, etc.). The challenge in developing LadeCA was therefore not to find or develop an optimal comparison function but to determine the optimal combination of comparison functions for each word. To the best of the author’s knowledge, the method for the flexible combination of comparison functions presented in “Comparison functions” section is the first of its kind, at least applied at this level of consistency. With this method, an individual classifier is automatically generated in real time for each word.

The method of adding new comparison functions to the existing base of comparison functions (Comparison functions section) to increase performance is also novel, and the performance of the new comparison functions can be compared with that of the existing base of comparison functions (with regard to selectable types of image and types of relatedness).

With two exceptions, the comparison functions used in LadeCA are standard methods. A brief overview is given in “Comparison functions” section. The two exceptions are the determination of characteristic areas in images using simulated fixations [28] and the detection and use of line elements as image features in addition to pixel information [7].

## Words in LadeCA

**Fig. 3:**
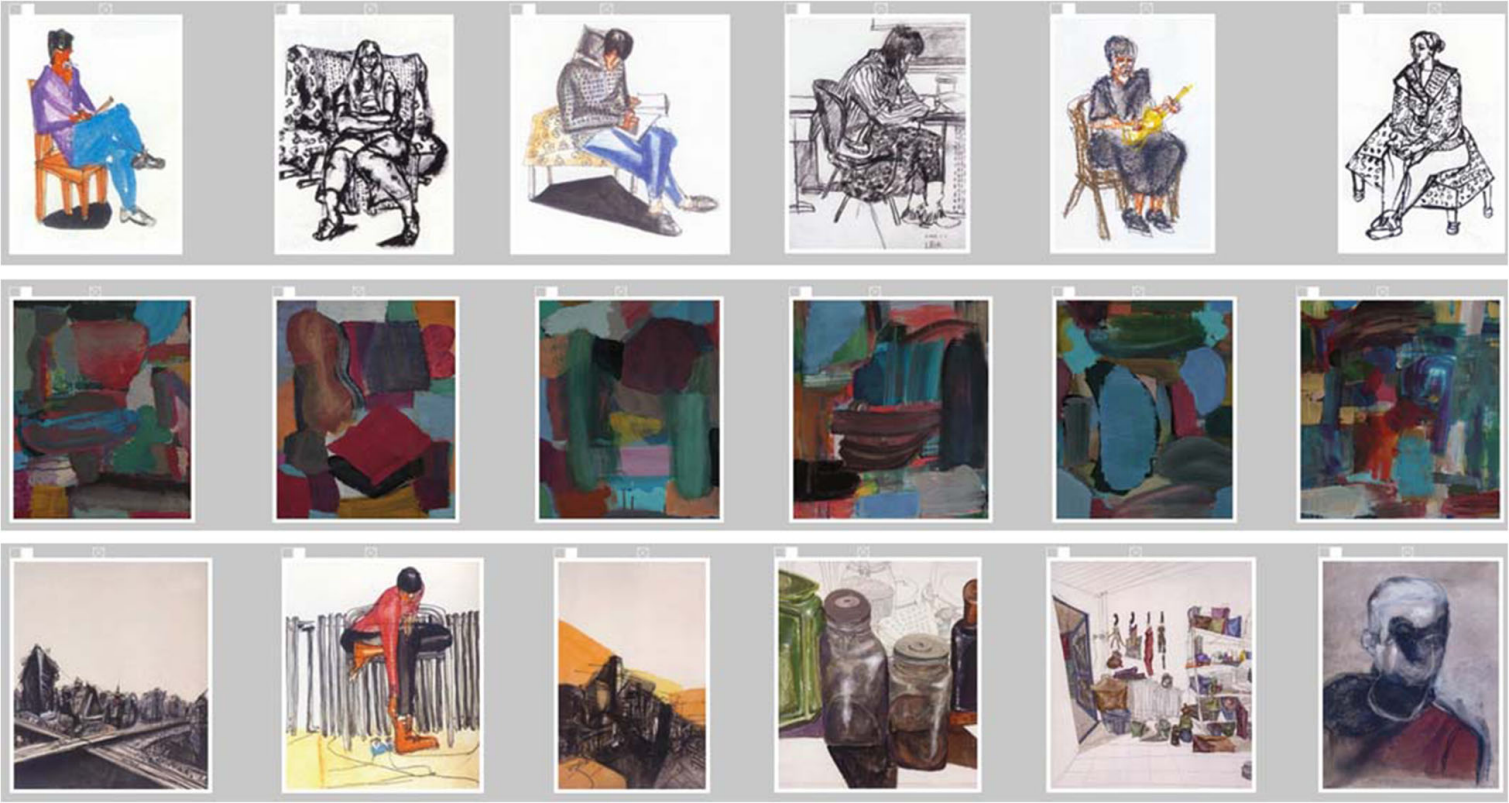
Different types of relatedness; artists from top to bottom: Weiran Wang, Christine Gläser, and Weiran Wang

A LadeCA word is determined based on related images. The types of relationships between the images of a word can be very different. Figure 3 shows three different types of relationships. In the top row, all the images show the same object - a seated person - and all the images are sketches. In the middle row, all the images are shaped by the artist’s style. In the bottom row, all the images are by the same artist with top row.

### Semiotic triangle

**Fig. 4:**
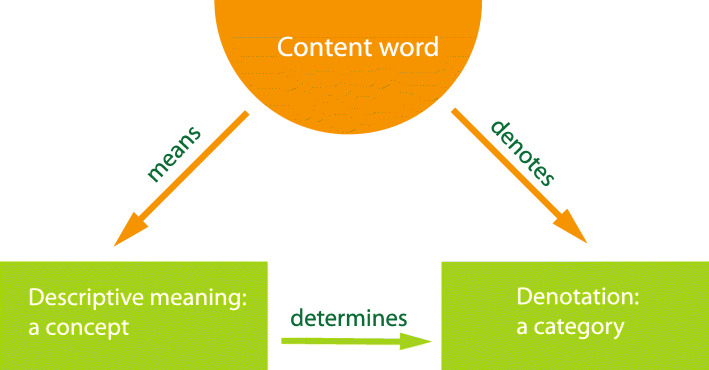
Semiotic triangle for a content word in general

The following describe the basic concept of a semiotic triangle for words of a natural language (Fig. 4) [23, 25]:

- A word is a sign expressed in spoken or written form.
- A word’s descriptive meaning is seen as a concept for potential referents. This concept is anchored in the individual experiences and thought patterns of the person using the word. In this sense, the descriptive meanings of a word are as manifold as the number of people using it. However, education and communication lead to words whose individual descriptive meanings converge to form a common descriptive meaning, called a lexical meaning, that enables both communication and semantic analysis.
- The denotation of a word is the intentional definition of potential referents or stated as the set of potential referents that can be labeled by the given word (extensional definition).

The semiotic triangle in LadeCA can be described as follows:

- A word is a sign expressed by an image or ideogram.
- A word’s descriptive meaning is a set of example images (possibly with metadata and text) that describe the meaning of the word. In this sense, the descriptive meanings are as manifold as the descriptive meanings in natural language, and it is assumed that usage or explicit stipulations also lead to a common descriptive meaning for each word.
- The denotation of a word is the set of potential images that can be labeled by the given word (extensional definition). Each word is associated with a unique classifier (intentional definition) that decides whether each given image can be labeled with the corresponding word or not.

### Word formation in LadeCA

**Fig. 5:**
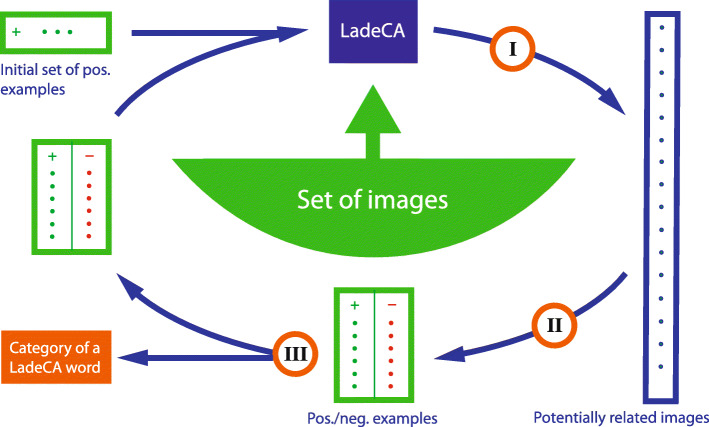
Schematic representation of the LadeCA word category formation process. Set of images: basis set used for the creation of classifiers. Initial set: one or more related images specified by the user or suggested by LadeCA based on analysis or clustering of the given set of images (or a preexisting LadeCA word). Step I: from the given set of positive and negative examples that describe a category (or the initial set), LadeCA creates a classifier, which in turn determines images potentially of that category. Step II: LadeCA separates the images generated in step I into those belonging/not belonging to the category, and the user makes corrections if necessary. Step III: if corrections have been made, a further iteration of the word category forming process is conducted

**Fig. 6:**
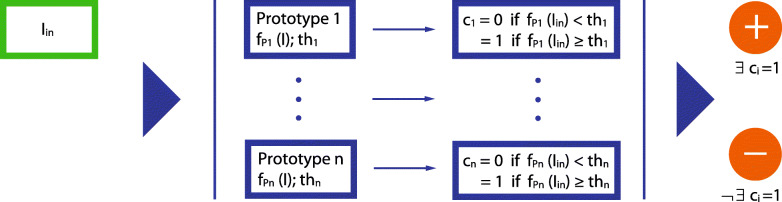
Schematic representation of the classifier of a category (Fig. 5). I_in_: any image to be classified. *n*: number of prototypes of the LadeCA word. f_Pi_: classification function of prototype *i*. t_hi_: threshold for classification. c_i_: classification result of I_in_ regarding prototype *i*

**Fig. 7:**
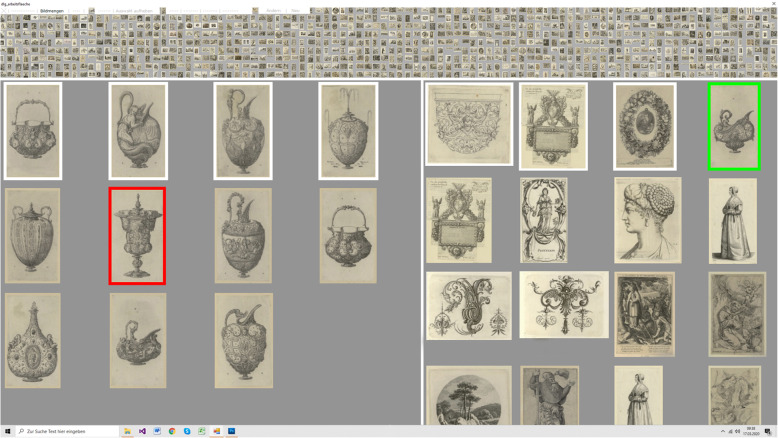
Interface for correcting a LadeCA word category

The interface for the formation of LadeCA words and the classifiers underlying these words play central roles in LadeCA. Nevertheless, both the design decisions for the interface and the choice of methods for the classifiers are beyond the scope of this study and are described in a separate paper. This paper is limited to describing the interface and outlining the principles according to which the classifiers are created. The semantic concept of LadeCA words follows prototype theory [23]. In prototype theory, a concept of a given language has a real-world example (prototype) that best represents this concept. Following this theory, a word can be defined by prototypes. Concepts in art include works of art that serve as real-world examples (prototypes). In this manner, LadeCA words are described through image examples, called prototypes, which represent the concepts underlying the words. In the field of art, the concepts that can be assigned to a category are not restricted and therefore vary. In most cases, it is necessary to describe a category with several prototypes, with each single prototype defining a concept of the category. The classifiers and associated comparison functions are generated automatically by LadeCA during the word formation process (Fig. 5). The basis set used to create the classifiers must be representative of the images to be examined or described with the words created. The word formation process starts with an existing LadeCA word or a set of positive example (prototype) images (together with metadata) that exemplarily describe the category of the word to be formed. Then, for each positive example, LadeCA generates a classifier that decides whether a given image is related to the positive example or not (Fig. 6). In the next step, all images of the basis set are classified, and the result is presented to the user for correction. Figure 7 shows the interface for the correction process. The left-hand side of the interface shows the images that are classified as positive; the order in which the images are shown corresponds to the strength of their relatedness to the category, and the images framed in white are the given positive examples. The right-hand side shows the images that are classified as negative; the order corresponds to the strength of their relatedness to the category, and the images framed in white are the given negative examples. The user can make corrections; if there are images that are not related to the category on the left-hand side (images classified as positive), the user can mark them as not related (with a red frame), and if there are images that are related to the category on the right-hand side (images classified as negative), the user can mark them as related (with a green frame). The correction does not have to be conducted completely, i.e., it is sufficient to correct only a few incorrectly classified images. In the next iteration, LadeCA accounts for the corrections to derive new classifiers. Because the images are sorted according to their degree of relatedness to the category, only the top rows need to be looked at when making corrections on the right-hand side because the classification is only questionable for these images. It is the responsibility of the user to decide whether all concepts that should be taken into account when creating a category are represented by the sample set. If a concept is not represented by the positive examples and if examples are not shown in the top rows of the right-hand side, further images that represent the missing concept - found by other means, e.g., via metadata - can be inserted into the sample set.

### Comparison functions

Each category of a LadeCA word has its own classifier, and this classifier itself consists of numerous classifiers (Fig. 6), one for each prototype defined for that category. The classifier of a prototype is determined by a series of comparison functions. The criteria for relatedness on which an individual comparison function is based are given implicitly by the corresponding comparison algorithm. Each of these comparison functions is weighted according to its importance for the characterization of the prototype. The sum of the results of the comparison functions indicates the relatedness of a given image to the corresponding prototype, and a threshold (t_hi_ in Fig. 6) defines the value above/below which an image is/is not to be assigned to the prototype.

**Fig. 8:**
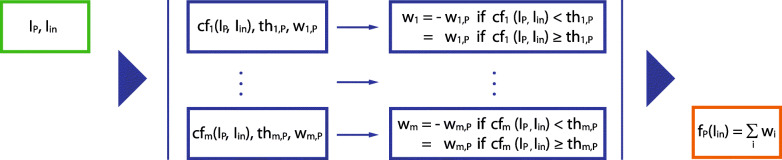
Schematic representation of the use of comparison functions (Fig. 6). I_P_: image of prototype *P* (together with its metadata). I_in_: any image to be classified. *m*: number of comparison functions given in LadeCA. cf_i_: comparison function *i*. th_i,P_: threshold of comparison function cf_i_ when used with prototype *P*. w_i,P_: weighting of the comparison function cf_i_ when used with prototype *P*. w_i_: result of the comparison function cf_i_ applied to images I_in_ and I_P_. f_P_: classification function of prototype *P*

The relatedness of two images is a very complex concept and is heavily dependent on the context and task; it cannot be determined by a universal method, i.e., with a single comparison function. Therefore, LadeCA currently has approximately 50 different comparison functions, which can be increased if new comparison functions are developed. Each LadeCA comparison function examines the relatedness between two images in terms of specific criteria or methods. The calculations in the execution of the functions can vary from being very simple to very elaborate and expensive, but all functions have two images as input and output a number between − 1 and + 1. The value − 1 indicates that the input images are completely different, and the value + 1 indicates that they are identical, in terms of both the criteria and method being used. An output value of a comparison function near 1 may be considered a kind of similarity measure (in terms of certain criteria) but does not represent a metric from a mathematical perspective. The optimal weights and thresholds (w_i,P_ and th_i,P_, respectively; Fig. 8) are calculated with Adaboost [29, 30] based on the positive and negative examples given by the user during the word formation process. To improve performance, the comparison functions usually do not use the pure pixel values of the input images; they use precalculated image features or given image information (such as metadata) instead. Therefore, in the context of LadeCA, an image consists not only of its pure pixel values but also of the total available image information; however, the nature and amount of information may vary. Accordingly, the input of the comparison functions consists of the pixel values of the images to be compared and all the available additional image information. If not all necessary information is available for a single classification for a specific comparison function, the weighting of the specific comparison function is set to 0, and the threshold value of the corresponding prototype is adjusted accordingly. This means that images that do not have all of the metadata used by a classifier can still be classified, which reduces the classification quality.

The comparison functions can be divided into three groups based on the types of relatedness they consider.

The first group uses image features to calculate relatedness. Image features are global features that describe an image as a whole [31] and convey the first impression an image makes on a viewer. These features can be, for example, color or brightness histograms or image properties, such as complexity or granularity. Image features can also be salient regions that are described by a standardized vector obtained by analyzing the corresponding region [32]. Each image feature spans a dimension in a feature space, and each image is defined by a single point in this space. It is assumed that if two images are located close to each other in the feature space, they are also similar, i.e., related, and under this assumption, comparison functions can be created via a metric defined in the feature space [33–35].

The second group uses given information about images to compare them in terms of relatedness. Most image databases provide such information as metadata, such as the names of the artists, image titles, years of origin, painting techniques, and sizes. Using this information, one can create suitable comparison functions. This can be done in a variety of ways, ranging from very simple to very complex. For example, the artist’s name can be used to build a comparison function. If two images are from the same artist, they may be considered related (return value + 1); if they are from different artists, they may be considered unrelated (return value − 1). One can also use the findings of art history to define different degrees of relatedness for different artists and thus to calculate a much more nuanced relatedness between two images, resulting in a return value from within the entire range − 1 to + 1. Metadata can be very helpful in comparing two images for relatedness. However, there are difficulties that limit their use. They are expensive to generate because they require expert knowledge and manual data input; any given type of metadata must have the same meaning and structure for all images used (from various databases); elaborate comparison functions require real (art) world knowledge for development.

The third group uses image properties that result from image elements and from their relationships to each other and to the overall image. The following procedure for a pair of images is representative of this type of comparison functions: (1) find salient areas, (2) perform matching, i.e., assigning the areas found for the two images to each other, and finally, (3) rate the matching, i.e., how well the salient areas are correlated. The idea behind this approach is that one can assume that similar generic constellations (contrast, rhythm, proportions, structures, etc. [11]) are given through similar and similarly arranged salient areas, and two images are related if they are formed by similar generic constellations. A typical representative procedure for this is the Sift algorithm [36–38]. Such commonly used methods are designed for object recognition and require too much computation time the purposes of LadeCA. Therefore, algorithms tailored especially to LadeCA were developed: algorithms similar to convolutional neural networks [28, 39] to determine characteristic areas in images and algorithms for matching these characteristic areas between two images. These methods are usually conducted pixel-wise, but it is also possible to detect lines in images and then use them for image comparisons [7, 40]. This option is used in several of LadeCA’s comparison functions to optimally consider line-based images (e.g., drawings and engravings).

The method for forming the classifier of a prototype, which uses the optimal comparison functions for recognizing the prototype to create its associated classifier, to the best of the author’s knowledge, is novel. Only in this way was it possible to create an interface for which users without computer science knowledge can create classifiers for the various contexts and types of artwork. Even if only comparison functions using visual image properties are considered, the resulting classifiers work well and are approximately equivalent for all types of artwork. For experiments including paintings (abstract, realistic, impressionistic, expressionistic, pointilistic, etc.) and drawings (charcoal, ink, etc.), we can see refs. [7, 28, 41–44]. Experiments that compare global image feature-based classifications with local image feature (and corresponding spatial distribution)-based classifications are reported in refs. [41, 43, 44] show that the performance of LadeCA’s algorithms is sufficient for interactive interfaces of the type described in this paper.

Although the methods and interfaces described in this paper can be used for any digital images, they are especially suited to images of visual art for the following reasons. Most of the comparison functions analyze salient regions in images. Everyday and technical images contain visual elements that are not significant to the image content. If these elements are visually conspicuous, then they affect the comparison functions negatively. Usually, artists paint major content in a distinctive and salient manner; therefore, regions with important content usually coincide with visually salient regions. Therefore, most of the comparison functions implicitly take particular account of the substantial image elements.

Another important property of the proposed method for creating classifiers is that the set of comparison functions can be easily expanded. For example, there are effective methods for detecting brushstrokes and hatchings, which can be used to compare images [40, 45–50]. If a new comparison function is created with these algorithms, then it can be very easily added to the existing comparison functions. Thereafter, the new comparison function will be considered when creating classifiers, i.e., when the new comparison function has advantages in terms of classification, it is used with a higher weight. A useful side effect is that by comparing the weighting factors, the efficiency of the new comparison function compared with that of an existing comparison function can be examined in terms of different types of images.

### Forming new words from existing words

Compounding: In LadeCA, there are three formal ways to create compound words from existing words: the union of two words A and B (A∪B) results in a new word that comprises all the images contained in A, B, or both; the intersection of two words A and B (A ∩ B) results in a new word that comprises all the images contained in both A and B; and the complement of a word A (¬A) results in a new word that comprises all images that are not contained in A. A and B may also be compound words. If a LadeCA user wants to edit a compound word, e.g., add more concepts or adapt the word to another database structure, then the compound word must be converted into a simple LadeCA word. To this end, LadeCA can create an initial set of positive image examples for the word formation process (Fig. 5). The user can then edit the compound word; in this manner, the compound word will be converted into a regular LadeCA word.

Deviation: It can be useful to vary LadeCA words to adapt them to specific tasks, e.g., to identify word fields or determine crossfades between two words. For this purpose, the threshold values of the classifiers (of the prototypes) and the comparison functions or weights of the comparison functions can be changed continuously. These changes in the parameters lead to the expansion or limitation of the number of images corresponding to a word.

Clustering: Words with a large number of associated images can be clustered [44]. The cluster process creates subsets of related images from the image set of a given word. These subsets can in turn be used to specify the given word by forming new words. This process can be automatic or user-controlled.

LadeCA can generate a word for a selected set of images from a base set; the classifier of this word then classifies strictly the selected images as positive. However, if the given set of images is not complete, i.e., if there exist images that actually belong to the word but are not in the selected set of images, the classifier of the formed word will not generalize accurately.

## Semantic relations

### Semantic relations of images

Semantic relations of images cannot be determined in an absolute manner. Whether two images belong together on a meaning level depends on the criteria according to which their relatedness is assessed, and these criteria are not static or generally applicable in the field of art (see examples in Fig. 3). In LadeCA, semantic relations of images can only be determined (automatically) and formulated through the prior selection and weighting of the available comparison functions. There are many ways to do this, which are outlined through the three following examples.

- **Example 1:**

The easiest way to compare images is to determine the words with which the images can be labeled. For example, if two images can be labeled with the same words, then either they can be seen as having the same meaning or the given vocabulary is not sufficient to adequately describe these images and should be expanded (Topological spaces section).

- **Example 2:**

To compare images, it is logical to use preexisting classification functions of the prototypes (Fig. 6). These functions indicate the relatedness of an image with a prototype by the simple means of a number. Values close to or greater than the threshold can be regarded as a measure of relatedness. The classification functions of a word span a vector space within which all images of the word can be located. In turn, this can be used to cluster the images of the word, with the individual clusters being a good template for breaking down the word into a finer structure.

- **Example 3:**

The most complex and expensive way to compare images is to optimize the weights and thresholds of the comparison functions specifically for the given task. For example, LadeCA determines the optimal weights and thresholds for each of the used comparison functions with Adaboost (Comparison functions section).

### Semantic relations of LadeCA words

Labeling an object/word does not clarify its meaning. To grasp the meaning of an object/word is to see it in relation to other objects/words [23–25, 51]. Therefore, a language about images must be able to indicate the relatedness between words. In LadeCA, there are two fundamentally different approaches to (automatically) examining the relatedness between words. On the one hand, word-based relatedness directly compares the word properties, i.e., using the classifiers and prototypes of the words. On the other hand, set-based relatedness uses a set of images (an exhibition, collection, or corpus) as a reference. Word-based relatedness allows more general statements, whereas the set-based relatedness adapts the algorithms that determine the relatedness of words to the given image material. It may be useful to use both approaches in parallel. If a collection of visual art is analyzed and the set-based relations are equal to the word-based relations, then the underlying collection confirms the general meanings of the words; otherwise, the collection has set out to question the words’ general meanings. Both word-based and the set-based approaches can be implemented in different ways, each with different strengths and weaknesses. Currently, LadeCA, uses one implementation for each of the word-based set-based approaches. The relatedness R between the words A and B is not symmetrical in either case (R_A,B_ ≠ R_B,A_). This allows an order (<) to be formulated, particularly to identify the property “A is a subset of B” (A ⊂ B).

In the word-based approach, the relatedness ${R}_{A,B}^W$ between words A and B is calculated by comparing all prototypes of word A with all prototypes of B using the classification functions of word B as follows:

$$ {\displaystyle \begin{array}{l}{R}_{A,B}^w=\left[{\sum}_i{\mathit{\operatorname{MAX}}}_j/\left(1-{th}_{B, PBj}\right)\right]/m\\ {}{R}_{B,A}^w=\left[{\sum}_j{\mathit{\operatorname{MAX}}}_i/\left(1-{th}_{A, PAi}\right)\right]/n\end{array}} $$

$$ {\displaystyle \begin{array}{l}{\mathit{\operatorname{MAX}}}_{\mathrm{j}}={\max}_j\left[{f}_{B, PBj}\left({P}_{Ai}\right)-{th}_{B, PBj}\right]\\ {}{\mathit{\operatorname{MAX}}}_i={\max}_i\left[{f}_{A, PAi}\left({P}_{Bj}\right)-{th}_{A, PAi}\right]\end{array}} $$

Here, *m* is number of prototypes of word A, *n* is number of prototypes of word B, *i* ∈{1, ..., *m*}; *j* ∈{1, ..., *n*}, *f*_A, PA*i*_ is classification function of word A and Prototype P_A*i*_ (Fig. 6), *f*_B, PB*j*_ is classification function of word B and Prototype P_B*j*_ (Fig. 6).

In the set-based approach, the relatedness ${R}_{A,B}^S$ between words A and B is determined by set theory as follows:

$$ {\displaystyle \begin{array}{l}{R}_{A,B}^S=\left|{I}_A\cap {I}_B\right|/\left|{I}_A\right|\\ {}{R}_{B,A}^S=\left|{I}_A\cap {I}_B\right|/\left|{I}_B\right|\end{array}} $$

|*I*_*A*_|(|*I*_*B*_|) is number of images of word A (B) in the reference set; |*I*_*A*_ ∩ *I*_*B*_| is number of images of the intersection of the images of the words A and B in the reference set.

Synonymy: The words A and B are considered strictly synonymous if both values R_A,B_ and R_B,A_ are equal to 1. In the word-based approach, this is the case if all prototypes of word A are classified as maximum-related to word B and vice versa. In the set-based approach, this is the case if the words A and B are identical with respect to the reference set, i.e., the images of word A in the reference set are the same as those of word B.

**Fig. 9:**
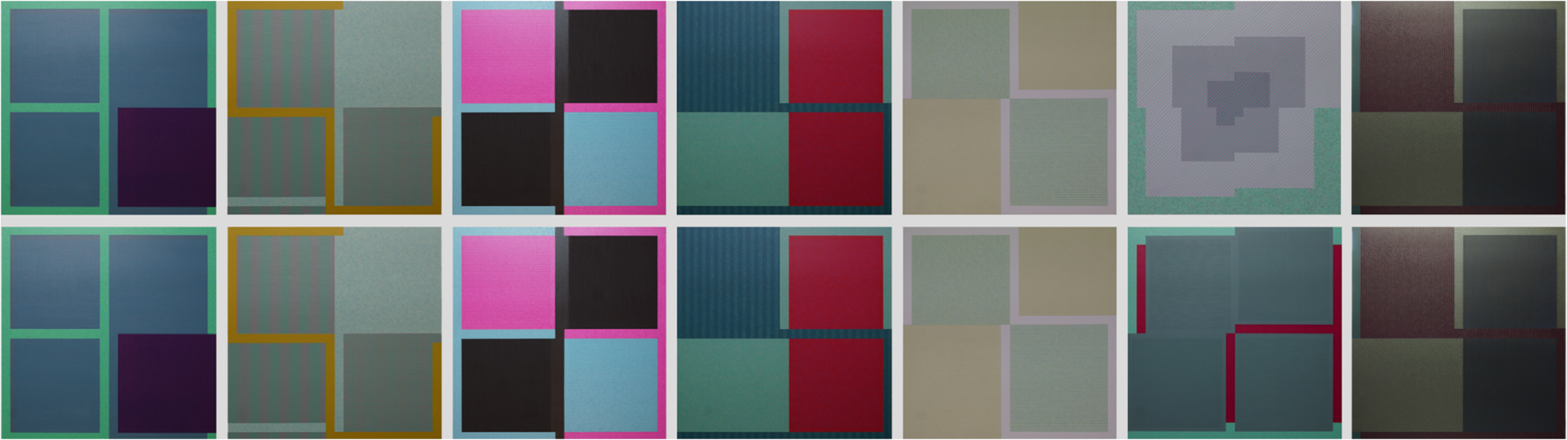
Example of two partially synonymous sets of images; artist: Frieder Kühner

Figure 9 shows an example of partial synonymy; the upper row is an image set partially synonymous with the image set in the lower row.

**Fig. 10:**
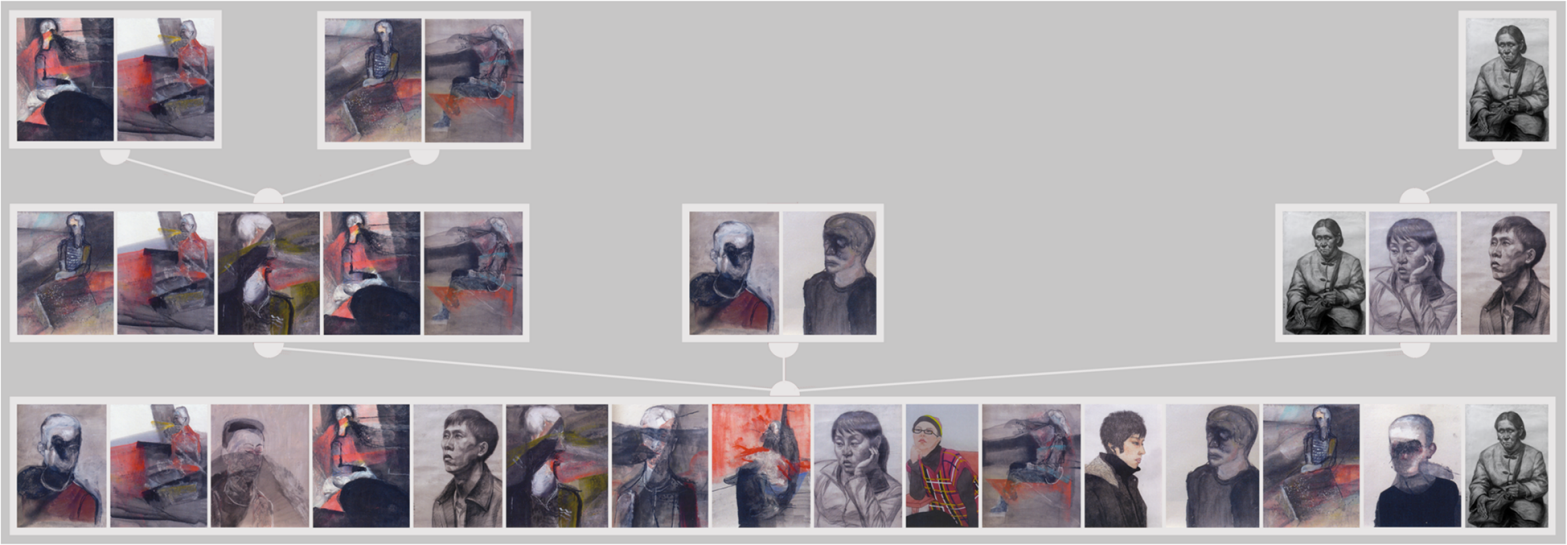
Examples of hyponymy; artist: Weiran Wang

Hyponymy: The word B is a strict hyponym of A (A is a hypernym of B) if R_B,A_ is equal to 1 and R_A,B_ is less than 1. In the word-based approach, this is the case if all prototypes of word B are classified as maximum-related to word A; however, the prototypes of word A are only partially represented by the prototypes of B. In the set-based approach, this is the case if the images of word B in the reference set compose a strict subset of the images of word A. Figure 10 shows examples of hyponymy; the sets of images in the middle row are hyponyms of the set in the bottom row, while they are hypernyms of the corresponding sets in the top row.

**Fig. 11:**
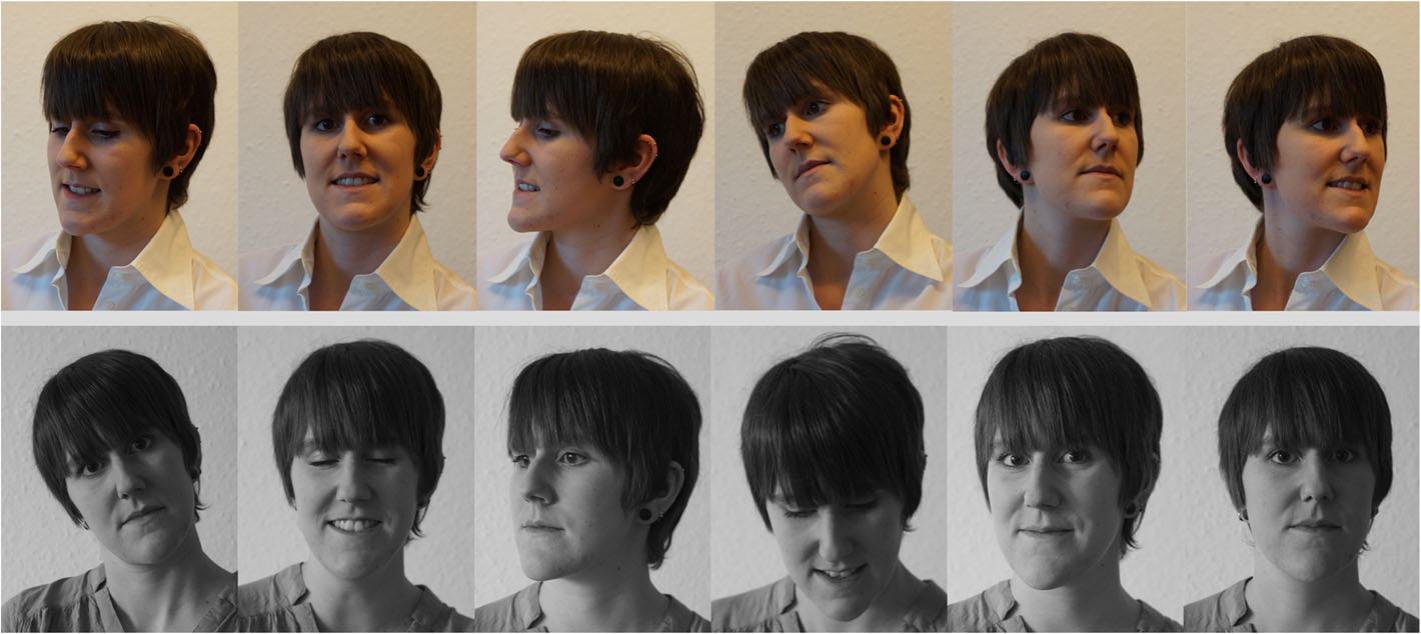
Example of co-hyponymy

Co-hyponymy: The words B_1_, B_2_, B_3_, etc., are strict co-hyponyms of A if R_B1,A_, R_B2,A_, R_B3,A_, etc., are equal to 1, R_A,B1_, R_A,B2_, R_A,B3_, etc., are less than 1, and the relatedness values R_Ai,Aj_ (with i ≠ j) are less than the corresponding thresholds (word-based) or 0 (set-based). In the word-based approach, this is the case if all prototypes of the words B_i_ are classified as maximum-related to word A and the words B_i_ are not related to each other. In the set-based approach, this is the case if the words B_i_ have no images in common and the images of word A are the exact sum of the images of words B_i_. Figure 11 shows an example of co-hyponymy; the images are divided into two independent parts: colored and gray-scale.

**Fig. 12:**
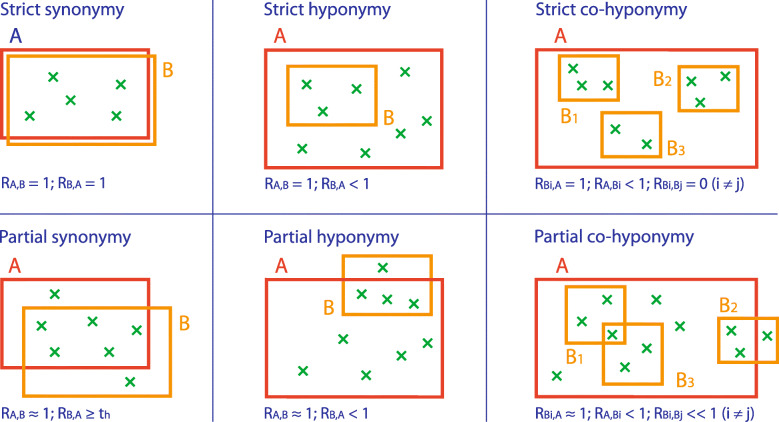
Euler diagrams showing synonymy, hyponymy, and co-hyponymy for the set-based approach

While strict synonymy, strict hyponymy, and strict co-hyponymy are rare or trivial, cases of partial synonymy, partial hyponymy, and partial co-hyponymy are more significant [23]. Figure 12 shows the difference between strict and partial synonymy, hyponymy, and co-hyponymy for the set-based approach; these differences apply analogously to the word-based approach.

Opposition/antonymy: When words B_1_ and B_2_ are strict co-hyponyms or partial co-hyponyms of a word A, they are opposites/antonyms or gradable antonyms, respectively.

Other methods for algorithmically determining word relatedness are given if complex expressions (structured image sets) are available or additional information is available via metadata (e.g., sets of indexed images). In LadeCA, it is generally true that word relatedness values that are significant in complex expressions or within a specific task and cannot be calculated algorithmically are always explicitly added to the words; there are no grammatical forms of LadeCA words.

## Results and discussion

A user study was conducted to evaluate the interface for word formation and determine the properties of LadeCA words. The study intended to clarify the following questions:

1. Can a LadeCA target group (in this study they are art historians) effectively handle the interface for generating LadeCA words?
2. How well can the target group distinguish LadeCA words, and how strong is the relationship between the distinctness of the words and calculated relatedness R?
3. Is it possible to create categories (Semiotic triangle section) in the field of visual arts and to define them with the help of image examples so that people can communicate using these image examples? In other words, is it possible for people to determine a category and describe it with sample images so that other people who see the sample images can understand the concept underlying the category?
4. The classifiers that belong to the LadeCA words are created based on a given image set (Fig. 5). Therefore, how accurately can the classifiers generalize, i.e., can the classifiers find the images that belong to the corresponding category in image sets other than the basis set?

**Fig. 13:**
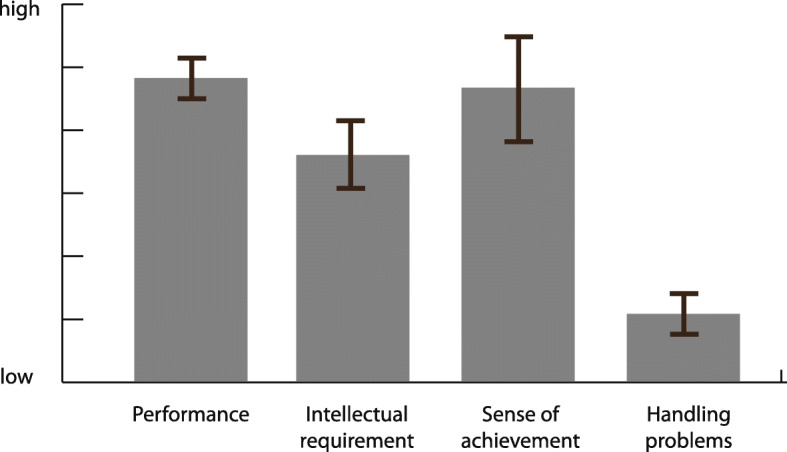
Subjective assessment of the participants as a result of the questionnaire (mean values with 95% confidence intervals)

Question 1: The study was conducted with 16 students from the Institute of Art History at the University of Stuttgart. All students had already attended an iconography lecture. As a basis for the study, the Albertina Vienna kindly provided 30,000 (scanned) historical prints from their collection (the *Klebealben*). This allowed not only the target group but also images that are relevant to art history and are usually used in this field to be considered. In the first part of the study, the participants formed LadeCA words based on 15,000 prints (the remaining 15,000 were used to evaluate the classifiers). For each word they created, they also had to describe the category of the word in a short text. After a short introduction of approximately 15 min, all participants were able to work on the task independently and required 5 to 10 min per word. This included finding a suitable category (with the given database functions), creating the word, and describing the category of the word in a short text. The results show that one of the system’s target groups managed to cope with the interface. At the end of the first part of the study, the participants filled out a questionnaire in which they subjectively assessed their performance and described their experience with the interface (Fig. 13).

**Fig. 14:**
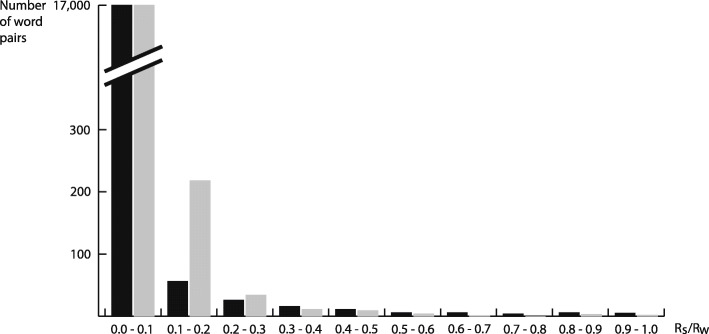
Number of word pairs divided into classes of the relation values *R*^*S*^ and *R*^*W*^ of all word pairs generated in the first part of the study. The black bars represent *R*^*S*^ values, and the gray bars represent *R*^*W*^ values

**Fig. 15:**
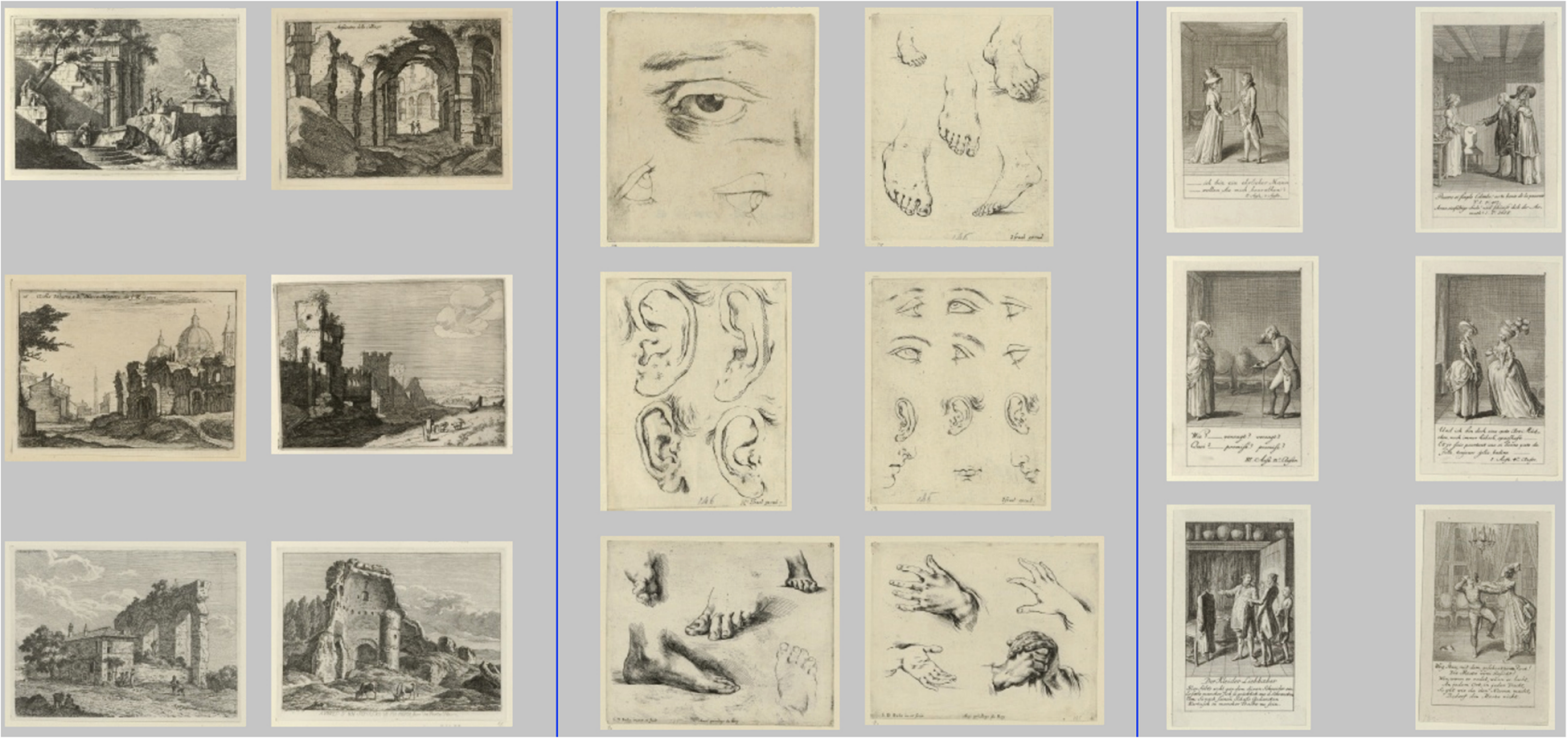
Image examples of three of the LadeCA words created in the study

To avoid any of the participants generating the same words, the images belonging to a corresponding word (maximum 40) were removed from the set of images after the word was created. The images were reinserted in the next part of the study. Figure 14 shows that most of the words were easily distinguishable using the relatedness *R*^*S*^ or *R*^*W*^. Nevertheless, there were some words that had a relatedness *R*^*S*^ or *R*^*W*^ greater than 0 because certain topics (e.g., portraits and landscapes) were very prevalent. The first part of the study culminated in sample images (a maximum of 18 images classified as positive; Fig. 15 shows three image examples) and a textual description for each of the 143 LadeCA words that the participants created. In the second part of the study, participants received the image samples of the words that were created by other participants in the first part and had to produce new textual descriptions. In addition, a further set of sample images was created for each word –images from the second set of images (another 15,000 images) classified as positive. For 11 words, there were insufficient images classified as positive in the second set (fewer than three images). Finally, the textual descriptions and image examples from the first part, further textual descriptions, and further descriptions via image examples (from the second set of images) were available for 132 words. In the third part of the study, each participant was given the two text-based descriptions and two descriptions with image examples of 10 words in a disorganized form. In addition, two text-based descriptions and two descriptions with image examples were inserted with no linkage among themselves or with the 10 given words. The task was, for the 10 given words, to assemble the different descriptions of each word. The number of correct and incorrect matches between text/text, text/original image examples, text/second set image examples, and original image examples/second set image examples were evaluated.

**Fig. 16:**
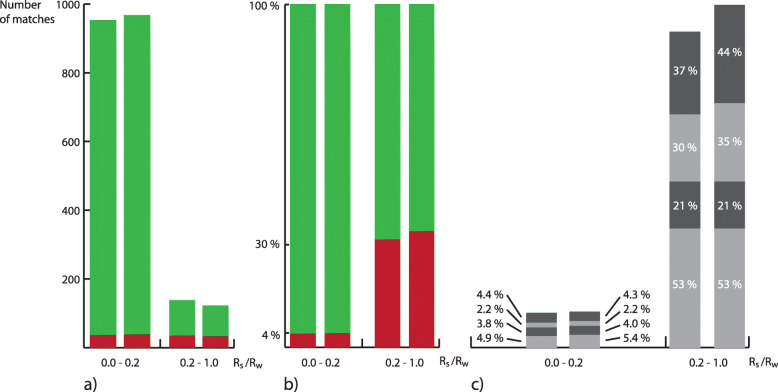
**a** Numbers of correct (green) and incorrect (red) matches. **b** Percentages of correct (green) and incorrect (red) matches. **c** Error rates of incorrect matches for (from bottom to top) text/text, text/original image examples, text/second set of image examples, and original image examples/second set of image examples. For the pairs of bars, the left bar always represents the *R*^*S*^ value and the right bar the *R*^*W*^ value

Question 2: To examine the distinguishability of the words, the matches from the third part of the study were evaluated. If the number of incorrect matches was small, the words were considered easy to distinguish, and Fig. 16a and b show a clear correlation between incorrect matching and the relatedness values *R*^*S*^ and *R*^*W*^. In the group with *R* values of less than 0.2, the error rate was less than 4%, whereas the error rate in the group with *R* values greater than 0.2 was approximately 30%; this indicates a strong correlation between the distinctness of the words and their relatedness. The text-based descriptions of the categories were considered to be the best available definitions for the individual words. Therefore, to more precisely assess the distinctness of the words given by the image examples, the matching text/text results were compared with the matching text/image results. The number of incorrect text/text matches (18 cases) was higher than that of the incorrect text/image example matches (9 cases). While the number of cases was too small to say that the description using image examples was more conclusive, this result confirms that description using image examples represents an equivalent alternative to textual description. It was also notable that in 7 of the 9 cases of incorrect text/image examples, the text/text and text/image example matches were also incorrect. It is concluded that in most cases of incorrect matching, either the textual description was insufficient, or the category of the word was generally difficult to define.

Question 3: Again, it was assumed that the text-based descriptions of the categories are the best available definitions for individual words. The text-based descriptions of the words created in the second part of the study were made with participants’ knowledge of the example images of the words but without knowledge of the original text-based descriptions. It was assumed that if the two text-based descriptions could be matched correctly in the third round, this would be an indication of the transferability of the category of the corresponding word with the help of the example images. There were 18 cases of incorrect text/text matches. In each of these cases, the reason for the mismatch was either a great similarity of the words (more precisely, of their example images) or that at least one of the descriptions was somehow lacking. Based on this assessment, sample images were assumed to usually be suitable for communicating categories of LadeCA words.

Question 4: In the first part of the study, the participants formed LadeCA words based on 15,000 historical prints. The formation process via the LadeCA interface ensured that the classifiers belonging to the words classified exactly the images of the basis set that belong to the corresponding words as positive. However, it is important to know how accurately the classifiers generalize. To investigate this, the classifiers of the words created in the first part of the study were applied to the remaining 15,000 unused images. This resulted in a new sample set of images for each of the created words. In the third part of the study, the participants were given these new sample sets of images and had to find the original words. It was assumed that if the participants were able to match the new sample sets to the corresponding words, the classifiers of each word generalized satisfactorily. The numbers of incorrect matches of the new example images with (1) the original example images (10 cases) and (2) the text-based descriptions (15 cases) were only slightly higher than that for the text-based descriptions with the original example images (9 cases; Fig. 16c). This indicates that the new image examples had almost the same information content as the original image examples, which in turn indicates very accurate generalization properties of the classifiers.

## Complex expressions in LadeCA

Words and their meanings form the basis of all semantic considerations. Besides knowing what a given word means in isolation, it is also important to know what words mean in the context of a given lexicon. This was discussed in “Words in LadeCA and Semantic relations” sections. Words are usually not uttered individually; they are combined into sentences. Sentence semantics (compositional meaning) deals with questions such as what role do words play in a sentence, how do their meanings intertwine, and what influence does the context in which a sentence is uttered have on its composition? [23] For a person to formulate complex statements, a language requires sentence formation and sentence semantics. It is currently not possible to state what options and demand there are to make complex statements using the LadeCA language; nevertheless, the following outlines approaches that may be suitable. Then, “Communications with LadeCA” section outlines ways in which such complex expressions can be created and communicated.

### Semantic networks

Here, a semantic network is considered to be a graph consisting of nodes that represent LadeCA words, or semantic fields, and edges that represent semantic relations among the nodes. Semantic networks have long been used in the field of linguistics [21, 22]. The novelty of LadeCA is the fact that semantic networks can be created automatically. Relationships between LadeCA words can be calculated automatically, and semantic fields can be determined automatically (Semantic relations section). Relations between semantic fields can also be defined in a similar manner and calculated algorithmically. There are many ways to design the properties of semantic networks and how they can be edited and visualized interactively. However, creating efficient interactive interfaces is very complex and only makes sense if the application is known. In “Communication via semantic networks” section, an interactive interface for the description of image collections is presented, which not only shows the words used in the collection as a semantic network but also the images assigned to the words. This interface can be used as a basis for further, more complex and specialized interfaces. All algorithms in LadeCA are sufficiently fast for semantic networks to be created, edited, and visualized interactively, even for large sets of images. Therefore, LadeCA has the potential to describe and visualize collections interactively in a wide variety of ways using semantic networks. For this reason, semantic networks in the context of LadeCA can be regarded as a means of describing collections of visual art analogous to sentences and texts in natural languages.

### Topological spaces

Here, discrete topologies (see refs. [52] or [53] for more information about topological spaces) that are formed from a vocabulary in connection with a set of images are investigated. In the context of this study, a topology is defined as follows:

- Each set of images belonging to a word (of the vocabulary) is an element of the topology.
- The intersection of two sets of the topology is an element of the topology.
- The union of two sets of the topology is an element of the topology.
- The complement of a set (all images of the base set that are not elements of the set) of the topology is an element of the topology.

The difference between a vocabulary and the topological space defined above is that this space contains not only the words of the vocabulary but also all possible combinations of words (Forming new words from existing words section). The elements of the topological space are consequently all the sets of images that result from a word or any combination of words applied to the given set of images. The author believes that the topological space defined here has great potential for the development of methods for analyzing and evaluating vocabularies and for analyzing and visualizing the properties of sets of images. The exact design of suitable tools and methods can only be determined using knowledge of the types of vocabularies, image sets, and associated questions and applications. The following two examples outline possible future directions of this approach.

The granularity with which an image set can be described by a vocabulary can be calculated using the topology described above. The smallest cover of an image in the topological space is the element of the topological space with the smallest cardinality that contains the given image, which means that the cardinality of the smallest cover of an image is the number of images that are described with the same combination of words. These images cannot be distinguished with the given vocabulary, which in turn indicates how accurately the images of the image set can be distinguished by the vocabulary. Thus, the distinctness calculated for each image in an image set defines the granularity of the vocabulary for this image set. A fine granularity means that the given set of images can be described very accurately by the vocabulary, whereas a coarse granularity either means that the image set contains many very similar images that cannot be differentiated further or that the vocabulary is not sufficiently powerful to describe each image appropriately and should thus be expanded. A vocabulary can be refined automatically or by a user (Comparison functions and Interaction between user and algorithms sections) in the areas where the vocabulary is too coarse for the given set of images.

The elements of the topology described above can be used as elements of a semantic network that can be treated analogously to a semantic network of words (Semantic networks section). The resulting network will be more subtle and conclusive than one consisting of words only, and each element of this network can be unambiguously defined with the given vocabulary using and/or operators.

### Indexing systems

The use of LadeCA words in the sense of words of a natural language presupposes that the individual concepts on which the LadeCA words are based converge towards a lexical meaning. Strictly speaking, a LadeCA word generated by an individual is only of importance to that individual. In other words, there are already two well-known systems of words in the field of visual art: ICONCLASS, a library classification, and AAT, a controlled vocabulary. The two systems differ in intent and conception, but both are used to index sets of visual art. If an image set is indexed, then the index terms are available as metadata and can be used for the automatic generation of LadeCA words (one word for each index term). The structure of the LadeCA words then corresponds to the structure of the indexing system in the context of the given image set, and all LadeCA interfaces (e.g., for analysis, visualization, and processing) can then be used in the meaning of the indexing system. LadeCA can then also be used to insert temporary keywords into the indexing system. Above all, a topology can then be generated from the LadeCA words, a topology with which the basic features of the indexing system (with reference to the given image set) can be examined, e.g., the granularity of the indexing system, which can then be used to suitably expand and improve the indexing system as a whole.

Systems such as ICONCLASS and AAT accompany basic difficulties that limit their use.

- Indexing must be done manually. Theoretically, it is possible to build a classifier for every element of a classification system, but this is not feasible in practice; an example set for each category (element of the system) would be necessary, considering all concepts (realizations) belonging to the category. In the field of art, the concepts that may be assigned to a category are not limited and are therefore varied and not static. For the foreseeable future, it would not be feasible to create a suitable sample set for each category of a classification system (such as ICONCLASS or AAT) and to train a classifier based on it. In addition, the corresponding classifier would have to be adapted for each new interpretation of a category. Manual indexing of all historic art is feasible if the task is divided among many people, and it seems safe to assume that most existing images are already indexed in a classification system. Nevertheless, there are still many images that are not indexed, and even more importantly, the number of images that must be classified for art history is increasing rapidly. Indexing currently unindexed and future images in a traditional manner would be very cost-intensive because it must be conducted by art experts.
- Another difficulty lies in the fact that even if experts perform the indexing, the assignment of images in a classification system is often ambiguous and therefore conducted differently by each expert. In addition, classification systems must be constantly adapted to new knowledge and requirements. Therefore, they are not static, and every change means that existing indexing must be checked - ultimately for all existing images.
- As a result of different requirements and tasks, there are various classification systems in use. Moreover, temporary systems or temporary extensions of existing systems would be desirable for special tasks. All of the above either makes multiple indexing operations necessary or leads to suboptimal classifications.

The difficulties listed above can be reduced by LadeCA. In “Interaction between user and algorithms” section, an interface that can significantly speed up and improve indexing is outlined. It is assumed that besides making it easier to index a set of images, as described above, an established indexing system and LadeCA can complement each other.

## Communications with LadeCA

Communication with LadeCA allows not only communication between people but also communication between people and algorithms (e.g., visual analytics) as well as the exchange of information between algorithms. If people are involved in the communication process, then communication occurs via interactive visual interfaces. In this section, two interfaces using LadeCA are introduced as examples of communication in which people are involved.

### Communication via semantic networks

The interface presented in this section is suitable for interactively creating abstracts of sets of images, showing one such abstract in an interactive visual manner; moreover, it is intended as a basis for more complex interfaces in the context of semantic fields of LadeCA.

**Fig. 17:**
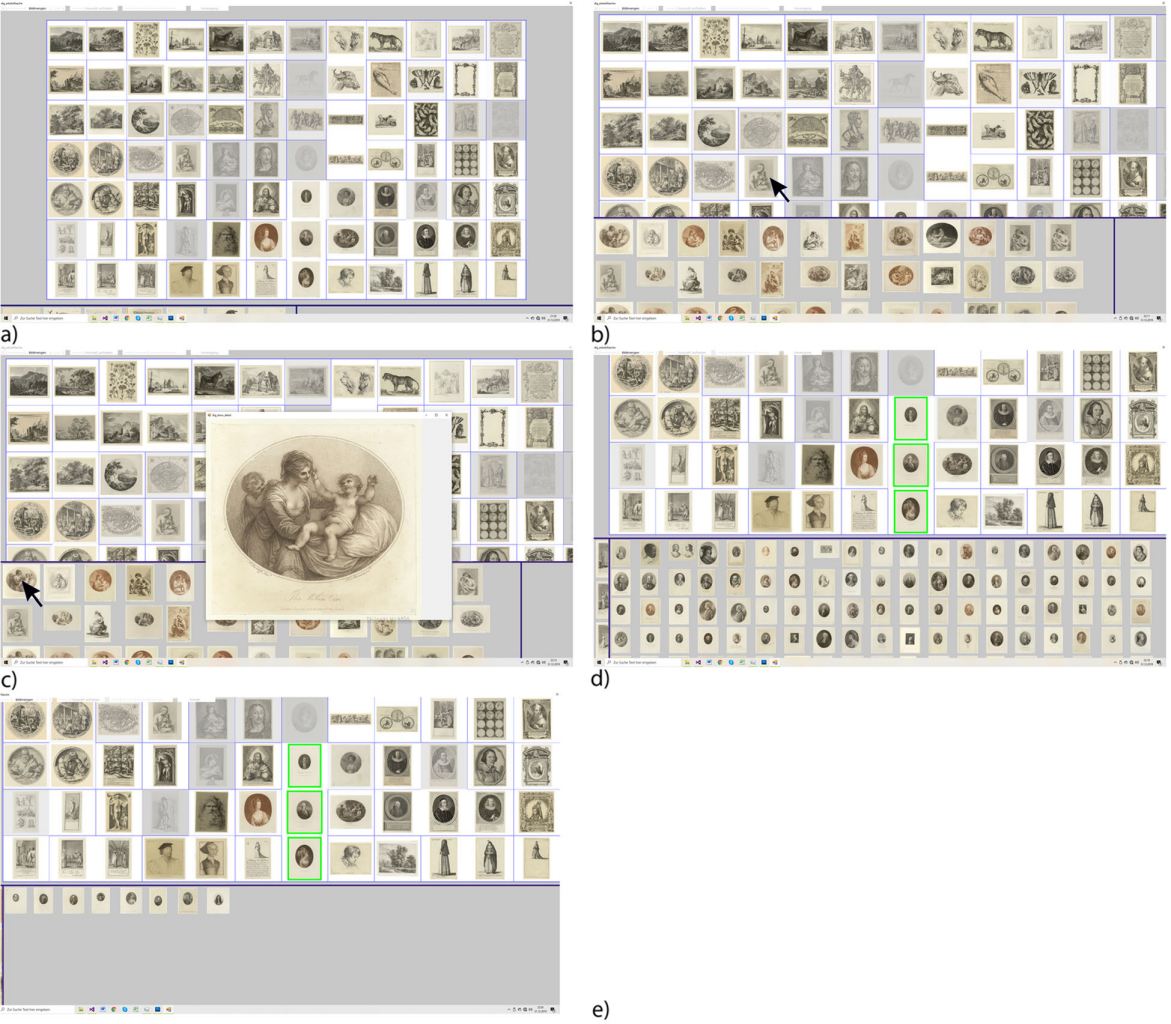
Screenshots of the interface for the presentation of an image set with a given vocabulary: **a** Vocabulary view maximized; **b** The bottom left view shows the images that are most closely related to the word closest to the mouse pointer; **c** Detailed information on the image nearest to the mouse pointer; **d** The bottom right view shows the images of the union of the words selected in the vocabulary view; **e** The bottom right view shows the images of the intersection of the words selected in the vocabulary view

The interface is divided into three linked views with manually adjustable sizes (Fig. 17). In all views, the user can pan and zoom. The topmost view (vocabulary view; Fig. 17a) is the main view and shows the representatives of all words. Words that are very strongly represented in the image set are shown as more opaque, while words for which only a few images exist in the image set are shown as more transparent. The system aims to arrange the words in such a way that words that appear next to each other are similar and/or of similar frequency in the set. The arrangement of the words is retained when moving and zooming, but the user can change the arrangement manually. The individual representatives are separated by blue lines. These lines indicate how closely the words are related. A thick line indicates that the two words are not related, whereas a thin or no line indicates strong relatedness. When the mouse pointer hovers over a word, the word is shown as fully opaque, and the 100 images most closely related to the word are displayed in the bottom left view ordered according to their relatedness to the hovered-over word (Fig. 17b). However, if the mouse pointer hovers over an image in the bottom left or bottom right view, details of the corresponding image (e.g., metadata or high-resolution details) are shown in a separate frame (Fig. 17c). If one or more words are selected in the vocabulary view, these words are marked (green), and in the bottom right view, either the union (Fig. 17d) or intersection (Fig. 17e) of these words is shown. The images in the bottom right view are sorted according to their relatedness to the corresponding word.

The example in Fig. 17 was generated with the 30,000 images from the user study and the words generated in the study (Results and discussion section). In this example, the interface runs on a standard PC without any significant delays. The vocabulary is approximately representative of the given set of images so that the presentation can actually be understood as an abstract. The interface can also be used as a filter; if the vocabulary represents only a certain type of image, e.g., only drawings, then the interface generates the representation of exactly this type of image taken from the given set of images. Therefore, a representation of the interface described here can be regarded as a linguistic formulation with the vocabulary as a statement in the context of the given set of images.

The design decisions resulting from the target group’s specifications (participants in the user study of this work and the study reported in ref. [7]). All images and representatives are shown side-by-side in their entirety [54] and not in an abstract manner, such as a point cloud [44]. Additional information is given separately [55] (blue lines that indicate relatedness) or in separate views or frames. The initial order of the words in the vocabulary view is calculated via a Kohonen map [56, 57].

The interface presented here can be considered as a platform for further developments towards more complex interfaces in the context of semantic networks of LadeCA. In future work, the interface will be expanded with further functions and methods as follows:

- Methods and functions with which a user can manually remove, change, or generate new words.
- Methods and functions that support a user in generating new words from existing words (Forming new words from existing words section).
- Methods and functions that support a user in generating and editing semantic fields (Semantic networks section).
- Visualizations to show the connections between words, between words and semantic fields, and between different semantic fields.
- Enhancement of the vocabulary view to make elements and structures of semantic fields accessible.

### Interaction between user and algorithms

**Fig. 18:**
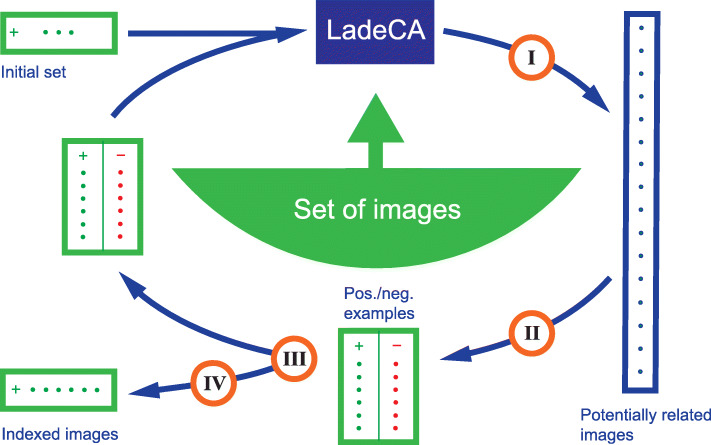
Indexing of categories instead of individual images using LadeCA. Set of images: a set of images to be indexed. Initial set: one or more related images specified by the user or suggested by LadeCA based on an analysis of the given set of images. Step I: based on the given set of positive and negative examples that describe a category (or the initial set), LadeCA creates a classifier, which in turn determines images potentially belonging to the category. Step II: supported by LadeCA, the user divides the given images as belonging/belonging to the category. Step III: the user decides whether the category defined by the positive images is complete. Step IV: the user indexes the category defined by the positive images

If an image set is indexed, the index terms are available as metadata and can be used for the automatic generation of LadeCA words (Forming new words from existing words section); each of these words then comprises exactly the images that were assigned a given index. In this manner, indexing systems can be integrated into LadeCA. The author is currently developing an interface that serves to enable users’ effective and fast indexing of image sets. With this interface, non-indexed image sets can be indexed, existing indexing can be improved, and the indexing system itself can be expanded or provided with temporary indexes. This interface is an example of how a user and algorithms can interact. Figure 18 schematically illustrates the work flow of the indexing process with the help of this interface.

The interface for indexing image sets is an extension of the interface for creating LadeCA words (Word formation in LadeCA section). The extension consists mainly of the formation process of the initial set at the beginning of each indexing step as well as the functions that support the indexing of a category. To generate the initial set, LadeCA first automatically creates a topology (Topological spaces section) from the words generated during the already completed indexing; if there is no indexing at the beginning, temporary indexes are created from existing LadeCA words and assigned to the images of the basis set. In the second step, the granularity of the existing indexing is calculated, and an image set is formed from the coarsest area by taking the images that cannot be distinguished by indexing. This set is then divided into several subsets by clustering (Forming new words from existing words section). Finally, one of these subsets is chosen as the initial set. The indexing steps are repeated until the desired indexing granularity has been achieved.

### Conclusion

This paper introduced the lexical base of LadeCA, a language that allows users to analyze, describe, and explore visual art. A user study was conducted, and the following results were obtained:

- The LadeCA target group can effectively handle the interface for generating LadeCA words.
- The target group can distinguish LadeCA words via image examples of the words; there is a strong relationship between the distinctness of the words perceived by the target group and the calculated relatedness of the words.
- It is possible to create categories in the field of visual arts and define them with the help of image examples so that people can communicate using these image examples.
- LadeCA word classifiers can generalize very accurately.

Moreover, this paper outlined how LadeCA words can be combined to form more complex expressions that can be used to analyze, describe, and explore collections of visual art. It also showed the relationship between LadeCA and indexing systems, such as ICONCLASS and AAT, and suggested ways in which LadeCA and indexing systems can complement each other.

The author is certain that LadeCA has great potential for analyzing, describing, and exploring large sets of visual art and is therefore looking forward to cooperating with LadeCA’s target groups to develop efficient and usable interfaces based on LadeCA.

## Supplementary Information

**Additional file 1: Video.**

## Data Availability

On demand.

## Abbreviation

LadeCA: A Language to analyze, describe, and explore collections of visual art
